# Acupuncture and Counselling for Depression in Primary Care: A Randomised Controlled Trial

**DOI:** 10.1371/journal.pmed.1001518

**Published:** 2013-09-24

**Authors:** Hugh MacPherson, Stewart Richmond, Martin Bland, Stephen Brealey, Rhian Gabe, Ann Hopton, Ada Keding, Harriet Lansdown, Sara Perren, Mark Sculpher, Eldon Spackman, David Torgerson, Ian Watt

**Affiliations:** 1 Department of Health Sciences, University of York, York, United Kingdom; 2 Centre for Health Economics, University of York, York, United Kingdom; 3 Hull York Medical School, University of York, York, United Kingdom; London School of Hygiene & Tropical Medicine, United Kingdom

## Abstract

**Background:**

Depression is a significant cause of morbidity. Many patients have communicated an interest in non-pharmacological therapies to their general practitioners. Systematic reviews of acupuncture and counselling for depression in primary care have identified limited evidence. The aim of this study was to evaluate acupuncture versus usual care and counselling versus usual care for patients who continue to experience depression in primary care.

**Methods and Findings:**

In a randomised controlled trial, 755 patients with depression (Beck Depression Inventory BDI-II score ≥20) were recruited from 27 primary care practices in the North of England. Patients were randomised to one of three arms using a ratio of 2∶2∶1 to acupuncture (302), counselling (302), and usual care alone (151). The primary outcome was the difference in mean Patient Health Questionnaire (PHQ-9) scores at 3 months with secondary analyses over 12 months follow-up. Analysis was by intention-to-treat.

PHQ-9 data were available for 614 patients at 3 months and 572 patients at 12 months. Patients attended a mean of ten sessions for acupuncture and nine sessions for counselling. Compared to usual care, there was a statistically significant reduction in mean PHQ-9 depression scores at 3 months for acupuncture (−2.46, 95% CI −3.72 to −1.21) and counselling (−1.73, 95% CI −3.00 to −0.45), and over 12 months for acupuncture (−1.55, 95% CI −2.41 to −0.70) and counselling (−1.50, 95% CI −2.43 to −0.58). Differences between acupuncture and counselling were not significant. In terms of limitations, the trial was not designed to separate out specific from non-specific effects. No serious treatment-related adverse events were reported.

**Conclusions:**

In this randomised controlled trial of acupuncture and counselling for patients presenting with depression, after having consulted their general practitioner in primary care, both interventions were associated with significantly reduced depression at 3 months when compared to usual care alone.

**Trial Registration:**

Controlled-Trials.com ISRCTN63787732

*Please see later in the article for the Editors' Summary*

## Abstract 2

In a randomized controlled trial, Hugh MacPherson and colleagues investigate the effectiveness of acupuncture and counseling compared with usual care alone for the treatment of depression symptoms in primary care settings.

*Please see later in the article for the Editors' Summary*

## Editors' Summary

**Background:**

Depression–overwhelming sadness and hopelessness–is responsible for a substantial proportion of the global disease burden and is a major cause of suicide. It affects more than 350 million people worldwide and about one in six people will have an episode of depression during their lifetime. Depression is different from everyday mood fluctuations. For people who are clinically depressed, feelings of severe sadness, anxiety, hopelessness, and worthlessness can last for months and years. Affected individuals lose interest in activities they used to enjoy and sometimes have physical symptoms such as disturbed sleep. Clinicians can diagnose depression and determine its severity by asking patients to complete a questionnaire (for example, the Beck Depression Inventory [BDI-II] or the Patient Health Questionnaire 9 [PHQ-9]) about their feelings and symptoms. The answer to each question is given a score and the total score from the questionnaire (“depression rating scale”) indicates the severity of depression. Antidepressant drugs are usually the front-line treatment for depression in primary care.

**Why Was This Study Done?:**

Unfortunately, antidepressants don't work for more than half of patients. Moreover, many patients would like to be offered non-pharmacological treatment options for depression such as acupuncture–a therapy originating from China in which fine needles are inserted into the skin at specific points of the body–and counseling–a “talking therapy” that provides patients with a safe, non-judgmental place to express feelings and emotions and that helps them recognize their capacity for growth and fulfillment. However, it is unclear whether either of these treatments is effective in depression. In this pragmatic randomized controlled trial, the researchers investigate the clinical effectiveness of acupuncture or counseling in patients with depression compared to usual care in primary care in northern England. A randomized controlled trial compares outcomes in groups of patients who are assigned to different interventions through the play of chance. A pragmatic trial asks whether the intervention works under real-life conditions. Patient selection reflects routine practice and some aspects of the intervention are left to the discretion of clinician, By contrast, an explanatory trial asks whether an intervention works under ideal conditions and involves a strict protocol for patient selection and treatment.

**What Did the Researchers Do and Find?:**

The researchers recruited 755 patients who had consulted their primary health care provider about depression within the past 5 years and who had a score of more than 20 on the BDI-II–a score that is defined as moderate-to-severe depression on this depression rating scale–at the start of the study. Patients were randomized to receive up to 12 weekly sessions of acupuncture plus usual care (302 patients), up to 12 weekly sessions of counseling plus usual care (302 patients), or usual care alone (151 patients). Both the acupuncture protocol and the counseling protocols allowed for some individualization of treatment. Usual care, including antidepressants, was available according to need and monitored in all three groups. Compared to usual care alone, there was a significant reduction (a reduction unlikely to have occurred by chance) in the average PHQ-9 scores at both 3 and 6 months for both the acupuncture and counseling interventions. The difference between the mean PHQ-9 score for acupuncture and counseling was not significant. At 9 months and 12 months, because of improvements in the PHQ-9 scores in the usual care group, acupuncture and counseling were no longer significantly better than usual care.

**What Do These Findings Mean?:**

These findings suggest that, compared to usual care alone, both acupuncture and counseling when provided alongside usual care provided significant benefits at 3 months in primary care to patients with recurring depression. Because this trial was a pragmatic trial, these findings cannot indicate which aspects of acupuncture and counseling are likely to be most or least beneficial. Nevertheless they do provide an estimate of the overall effects of these complex interventions, an estimate that is of most interest to patients, practitioners, and health care providers. Moreover, because this trial only considers the effect of these interventions on patients with moderate-to-severe depression as classified by the BDI-II; it provides no information about the effectiveness of acupuncture or counseling compared to usual care for patients with mild depression. Importantly, however, these findings suggest that further research into optimal treatment regimens for the treatment of depression with acupuncture and counseling is merited.

**Additional Information:**

Please access these Web sites via the online version of this summary at http://dx.doi.org/10.1371/journal.pmed.1001518.

- The US National Institute of Mental Health provides information on all aspects of depression (in English and Spanish)
- The UK National Health Service Choices website provides detailed information about depression, including personal stories about depression, and information on counseling and acupuncture
- The UK charity Mind provides information on depression, on talking treatments, and on complementary and alternative therapies including acupuncture; Mind also includes personal stories about depression on its website
- More personal stories about depression are available from Healthtalkonline
- MedlinePlus provides links to other resources about depression and about acupuncture (in English and Spanish)

## Introduction

Depression will be the second leading cause of disease burden worldwide by 2020, according to the Global Burden of Disease Study [1]. An estimated 2·6 million cases of depression were reported in England in 2000 [2]. It is the third most common reason for primary care consultations [3]. The economic burden is estimated to exceed UK£9 billion per annum in England, with direct costs of treatment being approximately UK£370 million [2]. Anti-depressants are the front-line treatment in primary care, yet an inadequate response to antidepressant treatment has been reported in up to 60% of patients [4] and lack of adherence to medication in 30% [3]. The effectiveness of newer anti-depressants for mild to moderate depression has recently come under question [5]. Patients have been concerned about an over-reliance on prescribed antidepressant medications and are keen to have access to non-pharmacological treatment options [6].

Acupuncturists commonly treat depression [7], but it is rarely provided within the UK's mental health services or primary care [8]. A Cochrane review found insufficient data on whether or not acupuncture is an effective intervention for depression, in part because of the high risk of bias in the majority of studies [9]. This review recommended the use of comparative designs in future research, comparing acupuncture to structured psychotherapies such as counselling or standard care. A widely used intervention for patients with depression is counselling, which is provided in approximately half of the 9,000 primary care practices in England [10]. Most counsellors provide a humanistic style of counselling. Recent National Institute for Health and Care Excellence (NICE) guidance is equivocal in recommending counselling for mild to moderate depression, identifying uncertainty regarding its effectiveness [11]. A Cochrane review, which identifies short-term but not long-term benefits from counselling, recommends extending the evidence base to include comparisons not just with usual care but also other interventions, including acupuncture, as this would have the potential to increase patient choice [12].

The primary aim of this study was to determine the clinical effectiveness and cost-effectiveness of short courses of either acupuncture or counselling when compared to usual care for patients with moderate to severe depression, with the secondary aim to compare acupuncture and counselling on the basis that there is structural equivalence regarding time and attention. The health economic results will be reported separately.

## Methods

### Ethics Statement

Ethical approval was obtained on 21st September 2009 from the York NHS Research Ethics Committee (ref: 09/H1311/75).

### Design

The design was that of an open parallel-arm randomised controlled trial with patients randomised by the York Trials Unit to one of three arms using the allocation ratio of 2∶2∶1, respectively: acupuncture plus usual care; counselling plus usual care; and usual care alone, as detailed in the published protocol [13]. Patient details were recorded by the Trials Unit prior to using Structured Query Language (SQL) software for computer-generated block randomisation, with block sizes of five and ten, conducted by an investigator with no clinical involvement in the trial. This ensured that the allocation was securely concealed from the researchers who subsequently informed patients of their allocation.

### Population

Patients who had consulted with depression in primary care within the past 5 years, and were continuing to experience moderate to severe depression were recruited. Identified from general medical practice databases, potential participants were aged 18 or over. Patients returned written consent forms and baseline questionnaires and were eligible if scoring 20 or above on the Beck Depression Inventory-II [14]. Thus all participants would be recognised as having depression. We used a different screening questionnaire from our chosen outcome measurement of depression, PHQ9 [15], because we did not want to use a screening questionnaire with a cut-off as the baseline measure in our analysis, to avoid problems of regression towards the mean. We did not want to give people the same questionnaire twice in fairly rapid succession, hence our choice of a second, also well-established, questionnaire for screening. We excluded patients who were receiving acupuncture or counselling at the time, who had terminal illness, significant learning disabilities, haemophilia, hepatitis, HIV, were pregnant, or who had confounding psychiatric conditions (bipolar disorder, postpartum depression, adjustment disorder, psychosis, dementia, or personality disorder). Patients who had suffered a close personal bereavement or given birth during the previous 12 months were excluded. Spoken English was a requirement.

### Interventions

Patients allocated to the acupuncture and counselling groups were offered up to 12 sessions usually on a weekly basis. Participating acupuncturists were registered with the British Acupuncture Council with at least 3 years post-qualification experience. An acupuncture treatment protocol was developed [16] and subsequently refined in consultation with participating acupuncturists. It allowed for customised treatments within a standardised theory-driven framework. Counselling was provided by members of the British Association for Counselling and Psychotherapy who were accredited or were eligible for accreditation having completed 400 supervised hours post-qualification. A manualised protocol, using a humanistic approach, was based on competences independently developed for Skills for Health [17]. Practitioners recorded in logbooks the number and length of sessions, treatment provided, and adverse events. Further details of the two interventions are presented in Tables S2 and S3. Usual care, both NHS and private, was available according to need and monitored for all patients in all three groups for the purposes of comparison.

### Outcome Measures

Our primary outcome measure was the PHQ-9 [15] at 3 months. In addition, we evaluated the overall impact over a 12-month period. PHQ-9 scores range from 0 to 27, with depression considered mild (5–9), moderate (10–14), moderately severe (15–19), or severe (20 and above). The EQ-5D was used as a preference-based measure of health outcome [18]. The use of medication was ascertained by asking patients whether they had taken any prescribed medication for depression or any prescribed analgesics/painkillers. Patients were asked to report health service use, including the number of times they had consulted a health professional because of their depression. Out-of-pocket costs included patient reports on spending on acupuncture, counselling, or psychotherapy including cognitive behavioural therapy. These data were collected at baseline and by postal questionnaire at 3, 6, 9, and 12 months These data were self-reported by patients independent of the research team, thereby avoiding problems of potential differentials between trial arms, such as unblinding, that could lead to bias. BDI-II was collected at baseline and 12 months. We collected baseline demographic data and patients' prior preferences and expectations of the interventions. For both acupuncture and counselling groups, we measured patients' perceptions of their practitioners' empathy at 3 months using the Consultational and Relational Empathy (CARE) measure [19]. A reimbursement of UK£5 was enclosed with the final questionnaire to enhance response rate [20]. In-depth interviews were used to explore the experiences and perspectives of patient and practitioner, the results of which will be reported separately.

### Sample Size

An effect size of 0·39 on the PHQ-9 was sought when comparing either acupuncture versus usual care alone or counselling versus with usual care alone. An allocation ratio of 2∶2∶1 was chosen in order to increase power to detect statistically significant differences between acupuncture and counselling. As a result, a smaller effect size of 0·32 was sought when comparing acupuncture and counselling. Anticipated differences between the treatments were likely to be smaller but not clinically meaningful. With 90% power and a two-sided significance level of 5%, the required group sizes were 204, 204, and 102 participants in the acupuncture, counselling, and usual care alone arms, respectively. Allowing for 20% attrition, the total sample size required was 640 (i.e., groups of 256, 256, 128, respectively).

### Analysis

The primary effectiveness comparisons were between acupuncture plus usual care versus usual care alone, and counselling plus usual care versus usual care alone. A secondary comparison was between acupuncture plus usual care versus counselling plus usual care. The primary outcome was PHQ-9 at 3 months, for which we used analysis of covariance (ANCOVA), with baseline PHQ-9 score as covariate. Multiple imputation by chained regression was used for missing data using treatment group, baseline measures (PHQ-9, BDI-II, SF-36, EQ-5D Anxiety/Depression), and demographics (age and gender). The primary analysis was based on the imputed rather than raw data in order to take account of the profile of non-responders.

To assess the overall clinical impact at 12 months, we evaluated area under the curve (AUC) for PHQ-9 over that period by linear regression, predicting average AUC while controlling for baseline PHQ-9. We also explored PHQ-9 outcomes across all time points in more detail using random intercept linear mixed models with fixed effects for treatment arm, time, and arm by time interaction for each treatment comparison. The models nested time points within patients and controlled for baseline PHQ-9 and potential mediators including patients' prior expectations and preferences regarding the treatments. These were identified by univariate regressions (*p*<0·1) of PHQ-9 at 3 months for the whole patient sample, controlling for baseline PHQ-9. When comparing acupuncture and counselling, we controlled for treatment time (combined length of sessions) and quality of attention (CARE empathy score). Treatment differences between BDI-II depression scores at 12 months were analysed by ANCOVA, controlling for BDI-II baseline scores and covariates for depression as identified above. Missing data were multiply imputed as for the PHQ-9.

All analyses were carried out in Stata (version 12.1) on an intention-to-treat basis. Statistical tests were at a two-sided 0·05 significance level. Analysis of residuals was undertaken for all regression models in order to assess model assumptions.

## Results

### Participants

**Figure 1:**
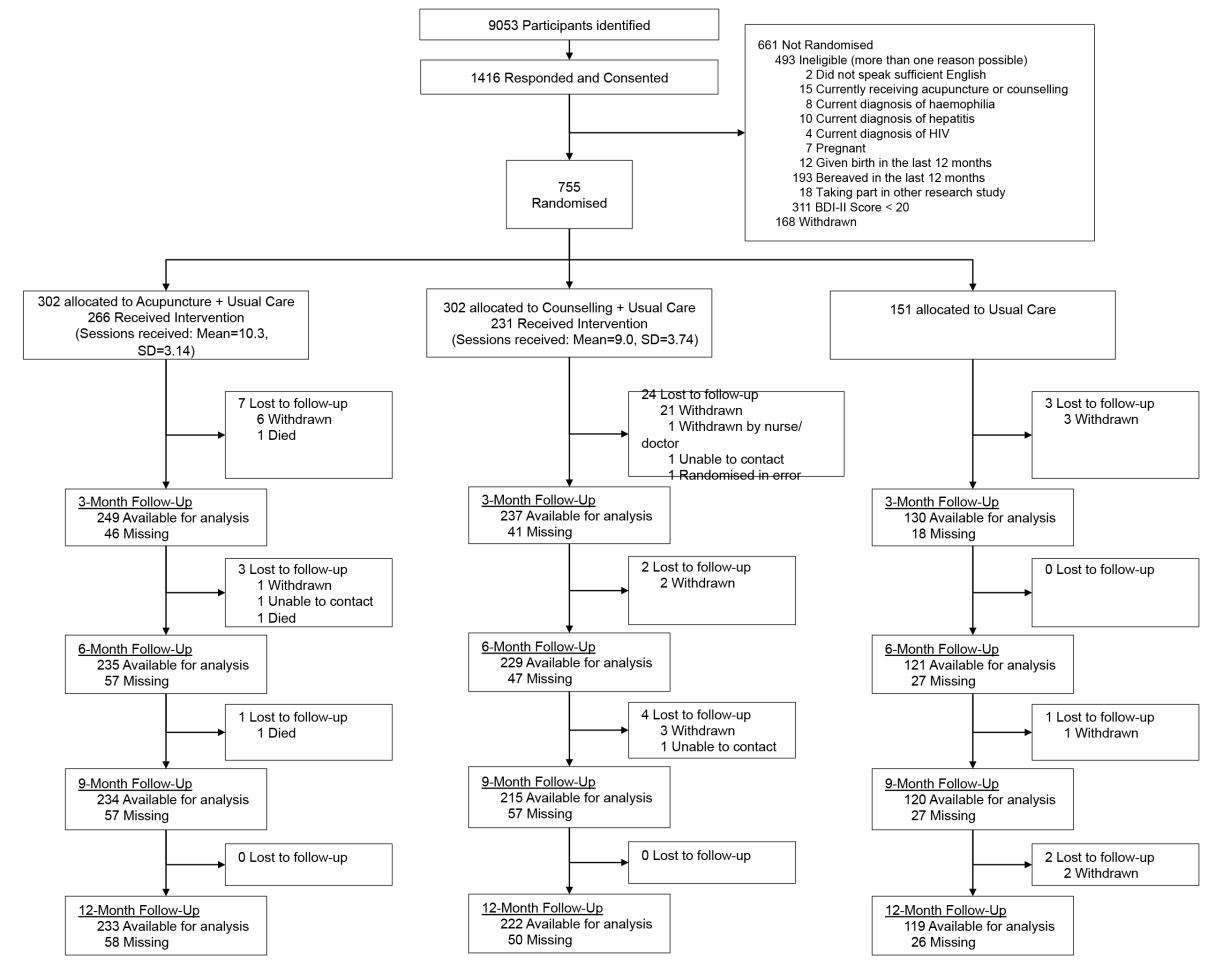
CONSORT flow diagram.

Patient flow in the trial is presented in Figure 1. In total 755 patients were recruited, 18% over target, from 27 general medical practices between December 2009 and April 2011 in Yorkshire and northeast England. General practices recruited an average of 28 patients (range 0 to 122). Baseline patient characteristics were balanced between the trial arms and are presented in Table 1. These characteristics included data on expectations of the treatments, which were lowest for acupuncture (68% were undecided) and preferences, for which the majority (58%) would have preferred to have been allocated acupuncture. These variations were taken into account in the linear mixed model analysis. Comparison of baseline data between patients with and without missing data at 3 months is presented in Table S1.

**Table 1:** Demographics and variables of interest at bastline.

Characteristic	Acupuncture + Usual Care	Counselling + Usual Care	Usual Care	Total
***n***	**302**	**302**	**151**	**755**
**Age**				
Mean (SD)	43·4 (13·24)	43·5 (13·26)	43·5 (13·93)	43·5 (13·37)
Median (min, max)	43 (18–86)	43 (18–93)	42 (18–89)	43 (18–93)
Inter-quartile range	34–52	33–52	32–54	33–53
Missing	–	–	–	–
**Sex**				
Male	88 (29·1%)	69 (22·9%)	44 (29·1%)	201 (26·6%)
Female	214 (70·9%)	233 (77·1%)	107 (70·9%)	554 (73·4%)
Missing	–	–	–	–
**Age left education**				
Mean (SD)	18·0 (4·69)	18·0 (3·92)	18·1 (4·62)	18·0 (4·37)
Median (min, max)	16 (13–54)	16 (14–48)	16 (14–54)	16 (13–54)
Inter-quartile range	16–18	16–19	16–19	16–19
Missing	17 (5·6%)	7 (2·3%)	7 (4·6%)	31 (4·1%)
**Employment**				
Full-time education	13 (4·4%)	5 (1·7%)	5 (3·3%)	23 (3·1%)
Working full-time	112 (38·0%)	107 (36·4%)	62 (41·3%)	281 (38·0%)
Working part-time	57 (19·3%)	59 (20·1%)	28 (18·7%)	144 (19·5%)
Unable to work	38 (12·9%)	42 (14·3%)	15 (10·0%)	95 (12·9%)
Looking after home	37 (12·5%)	32 (10·9%)	14 (9·3%)	83 (11·2%)
Retired	23 (7·8%)	30 (10·2%)	12 (8·0%)	65 (8·8%)
Other	15 (5·1%)	19 (6·5%)	14 (9·3%)	48 (6·5%)
Missing	7 (2·3%)	8 (2·6%)	1 (0·7%)	16 (2·1%)
**Depression**				
In last 2 wk	224 (75·7%)	235 (78·6%)	115 (77·7%)	574 (77·3%)
Missing	6 (2·0%)	3 (1·0%)	3 (2·0%)	12 (1·6%)
Not first major episode	196 (89·5%)	217 (93·5%)	100 (87·7%)	513 (90·8%)
Missing	5 (2·2%)	3 (1·3%)	1 (0·9%)	9 (1·6%)
4+ previous episodes	143 (73·0%)	165 (76·7%)	81 (82·7%)	389 (76·4%)
Missing	0 (0·0%)	2 (0·9%)	2 (2·0%)	4 (0·8%)
**Age at first major depressive episode**				
Mean (SD)	25·8 (12·69)	24·9 (11·73)	24·4 (12·55)	25·2 (12·28)
Median (min, max)	23 (3–79)	22 (6–71)	20 (0–78)	22 (0–79)
Inter-quartile range	16–33	16–31	16–30	16–31
Missing	9 (3·0%)	3 (1·0%)	4 (2·6%)	16 (2·1%)
**Medication**				
Depression medication in last 3 mo	189 (62·6%)	220 (72·9%)	110 (72·9%)	519 (68·7%)
Missing	0 (0·0%)	0 (0·0%)	0 (0·0%)	0 (0·0%)
Analgesic medication in last 3 mo	147 (48·8%)	126 (42·3%)	86 (57·3%)	359 (47·9%)
Missing	1 (0·3%)	4 (1·3%)	1 (0·7%)	6 (0·8%)
**EQ-5D Anxiety/Depression**				
Not anxious/depressed	8 (2·7%)	8 (2·7%)	5 (3·3%)	21 (2·8%)
Moderately anxious/depressed	219 (73·0%)	221 (73·2%)	114 (75·5%)	554 (73·6%)
Extremely anxious/depressed	73 (24·3%)	73 (24·2%)	32 (21·2%)	178 (23·6%)
Missing	2 (0·7%)	0 (0·0%)	0 (0·0%)	2 (0·3%)
**PHQ-9**				
Mean (SD)	15·3 (5·33)	16·6 (5·27)	16·2 (5·09)	16·0 (5·29)
Median (min, max)	15 (3–27)	17 (4–27)	16 (5–27)	16 (3–27)
Inter-quartile range	11–19	13–21	13–20	12–20
Missing	1 (0·33%)	0 (0·0%)	0 (0·0%)	1 (0·1%)
**PHQ Group**				
None (0–4)	4 (1·3%)	2 (0·7%)	0 (0·0%)	6 (0·8%)
Mild (5–9)	44 (14·6%)	29 (9·6%)	14 (9·3%)	87 (11·5%)
Moderate (10–14)	97 (32·2%)	74 (24·5%)	46 (30·5%)	217 (28·8%)
Moderately severe (15–19)	88 (29·2%)	96 (31·8%)	47 (31·1%)	231 (30·6%)
Severe (20–27)	68 (22·6%)	101 (33·4%)	44 (29·1%)	213 (28·3%)
**BDI-II**				
Mean (SD)	32·0 (8·54)	33·3 (9·11)	31·8 (8·17)	32·5 (8·72)
Median (min, max)	31 (20–57)	32 (20–60)	30 (20–56)	31 (20–60)
Inter-quartile range	25–37	26–39	25–37	26–38
Missing	–	–	–	–
**BDI-II Group**				
Moderate (20–28)^2^	124 (41·1%)	104 (34·4%)	56 (37·1%)	284 (37·6%)
Severe (29–63)	178 (58·9%)	198 (65·6%)	95 (62·9%)	471 (62·4%)
**SF-36 Bodily Pain**				
Mean (SD)	58·8 (27·99)	58·0 (29·17)	54·4 (27·83)	57·6 (28·44)
Median (min, max)	62 (0–100)	62 (0–100)	51 (0–100)	52 (0–100)
Inter-quartile range	41–84	31–84	31–74	32–84
Missing	1 (0·3%)	3 (1·0%)	0 (0·0%)	4 (0·5%)
**Expectation acupuncture**				
Very ineffective	10 (3·3%)	6 (2·0%)	1 (0·7%)	17 (2·3%)
Fairly ineffective	9 (3·0%)	11 (3·7%)	6 (4·0%)	26 (3·5%)
Can't decide	187 (61·9%)	204 (68·0%)	103 (68·2%)	494 (65·6%)
Fairly effective	66 (21·9%)	44 (14·7%)	29 (19·2%)	139 (18·5%)
Very effective	30 (9·9%)	35 (11·7%)	12 (8·0%)	77 (10·2%)
Missing	0 (0·0%)	2 (0·7%)	0 (0·0%)	2 (0·3%)
**Expectation counselling**				
Very ineffective	23 (7·7%)	18 (6·0%)	15 (9·9%)	56 (7·5%)
Fairly ineffective	50 (16·7%)	43 (14·3%)	21 (13·9%)	114 (15·2%)
Can't decide	101 (33·8%)	100 (33·2%)	39 (25·8%)	240 (32·0%)
Fairly effective	95 (31·8%)	97 (32·2%)	65 (43·1%)	257 (34·2%)
Very effective	30 (10·0%)	43 (14·3%)	11 (7·3%)	84 (11·2%)
Missing	3 (1·0%)	1 (0·3%)	0 (0·0%)	4 (0·5%)
**Expectation usual care**				
Very ineffective	29 (9·6%)	34 (11·3%)	6 (4·1%)	69 (9·2%)
Fairly ineffective	60 (19·9%)	62 (20·7%)	44 (29·7%)	166 (22·1%)
Can't decide	108 (35·8%)	92 (30·7%)	50 (33·8%)	250 (33·3%)
Fairly effective	95 (31·5%)	97 (32·3%)	46 (31·1%)	238 (31·7%)
Very effective	10 (3·3%)	15 (5·0%)	2 (1·4%)	27 (3·6%)
Missing	0 (0·0%)	2 (0·7%)	3 (2·0%)	5 (0·7%)
**Expectation of actual treatment randomised to**				
Very ineffective	10 (3·3%)	18 (6·0%)	6 (4·1%)	34 (4·5%)
Fairly ineffective	9 (3·0%)	43 (14·3%)	44 (29·7%)	96 (12·8%)
Can't decide	187 (61·9%)	100 (33·2%)	50 (33·8%)	337 (44·9%)
Fairly effective	66 (21·9%)	97 (32·2%)	46 (31·1%)	209 (27·8%)
Very effective	30 (9·9%)	43 (14·3%)	2 (1·4%)	75 (10·0%)
Missing	0 (0·0%)	1 (0·3%)	3 (2·0%)	4 (0·5%)
**Treatment preference**				
Acupuncture	177 (58·8%)	171 (57·6%)	82 (54·7%)	430 (57·5%)
Counselling	55 (18·3%)	75 (25·3%)	34 (22·7%)	164 (21·9%)
Usual Care	2 (0·7%)	7 (2·4%)	1 (0·7%)	10 (1·3%)
No preference	67 (22·3%)	44 (14·8%)	33 (22·0%)	144 (19·3%)
Missing	1 (0·3%)	5 (1·7%)	1 (0·7%)	7 (0·9%)
**Treatment concordance**				
Randomised to preferred treatment	177 (58·8%)	75 (25·3%)	1 (0·7%)	253 (33·8%)
Randomised to non-preferred treatment	57 (18·9%)	178 (59·9%)	116 (77·3%)	351 (46·9%)
No preference	67 (22·3%)	44 (14·8%)	33 (22·0%)	144 (19·3%)
Missing	1 (0·3%)	5 (1·7%)	1 (0·7%)	7 (0·9%)

Percent given out of non-missing data, percent missing given out of applicable group total.

A BDI-II score of 20 or above was required to be eligible for the trial.

### Interventions

In the acupuncture arm, 266 patients (88.1%) received one or more treatment sessions (mean  = 10.3, standard deviation [SD] 3.14) with one of 23 acupuncturists. By the 3-month time point, the average number of sessions received was 8.7 (SD = 3.34), with 133 patients (50.0%) having completed all of their sessions. In the counselling arm, 231 patients (76.5%) received one or more treatment sessions (mean  = 9.0, SD = 3.74) with one of 37 therapists. By the 3-month time point, the average number of sessions received was 7.5 (SD = 3.60), with 114 patients (49.4%) having completed all of their sessions. The mean time from randomisation to last treatment was 117 days in both treatment arms (SD = 47.0 and 51.2, respectively), a period that included time to first appointment. Acupuncturists were allocated an average of 13 patients each (range two to 45), and counsellors an average of eight patients each (range one to 27).

In the logbooks, we collected data on acupuncturists' and counsellors' self-reports of intervention protocol violations. We received reports of four cases of violations of the acupuncture protocol (which incidentally did not involve counselling), one of which was deemed a true case involving prescription of a herbal lotion application to reduce swelling and pain. We received seven reports of violations of the counselling protocol, of which two were deemed true cases; one case involved goal setting and in another the counsellor reported being analytical and interpretative beyond the scope of humanistic counselling.

Details of medication use over the period of the trial is reported below, and further details of all three interventions are summarised for acupuncture (Table S2) and counselling (Table S3), and, respectively, and for usual care: patients seeing a health professional (Table S4); patients attending hospital accident and emergency departments (Table S5); patients admitted to hospital (Table S6); and patients paying for private health care (Table S7). A more complete report on the acupuncture provision has now been published [21] and one on the counselling provision will follow shortly.

### Clinical Outcomes

**Figure 2:**
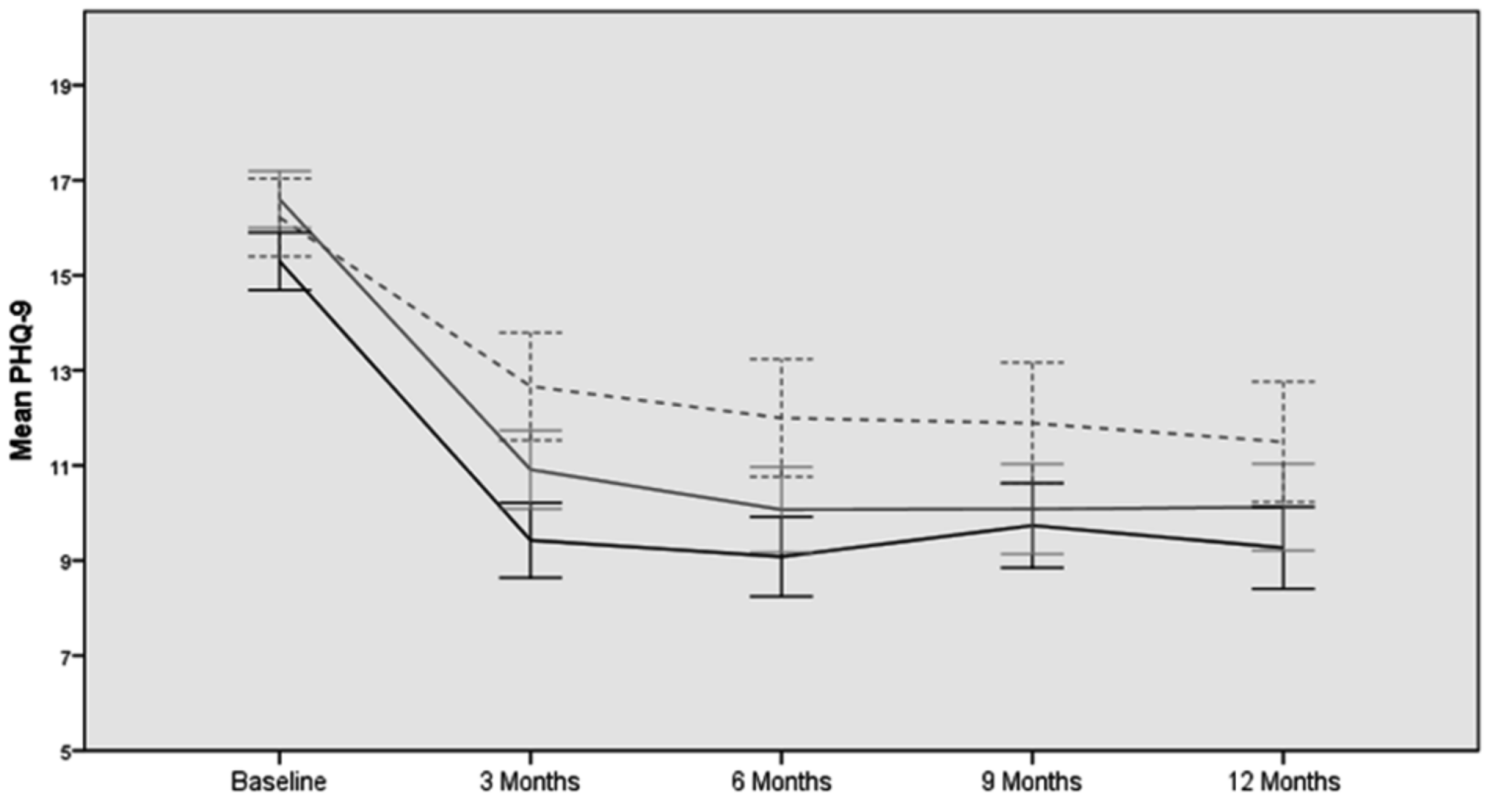
Unadjusted PHQ-9 mean scores from baseline to 12 months. Acupuncture, black line; counselling, grey line; usual care, dashed line.

Unadjusted mean scores for the PHQ-9 at all time points are presented in Table 2 and Figure 2. For the primary outcome, Table 3 gives results for between-group differences in PHQ-9 depression at 3 months using ANCOVA. Patients for whom no data were available at that point tended to be younger with higher levels of baseline depression, and imputed data were used to take their profile into account. Compared to usual care, patients in the acupuncture arm experienced an average additional reduction in depression of −2·46 points on the PHQ-9 (*p*<0·001, 95% CI −3·72 to −1·21), an observed effect size equivalent to a Cohen's *d* of −0·39 (95% CI −0·58 to −0·19). Compared to usual care, patients allocated to the counselling arm experienced an average additional reduction in depression of −1·73 points (*p* = 0·008, 95% CI −3·00 to −0·45), equivalent to a Cohen's *d* of −0·27 (95% CI −0·47 to −0·07). The difference between acupuncture and counselling, −0·76 points on the PHQ-9 (*p* = 0.41, 95% CI −1·77 to 0·25), was not statistically significant. These data are conservative, as non-imputed data showed slightly larger treatment effects (see Table S8).

**Table 2:** Unadjusted depression outcomes.

Outcome Measures	Acupuncture + Usual Care		Counselling + Usual Care		Usual Care		Total	
	*n*	Mean (SD)	*n*	Mean (SD)	*n*	Mean (SD)	*n*	Mean (SD)
PHQ-9								
Baseline	301	15·3 (5·33)	302	16·6 (5·27)	151	16·2 (5·09)	754	16·0 (5·29)
3 mo	249	9·4 (6·33)	237	10·9 (6·45)	128	12·7 (6·47)	614	10·7 (6·51)
6 mo	235	9·1 (6·51)	228	10·1 (6·87)	120	12·0 (6·85)	583	10·1 (6·80)
9 mo	234	9·7 (6·90)	215	10·1 (7·03)	120	11·9 (7·04)	569	10·3 (7·02)
12 mo	233	9·3 (6·68)	220	10·1 (6·86)	119	11·5 (6·98)	572	10·1 (6·85)
BDI-II								
Baseline	302	32·0 (8·54)	302	33·3 (9·11)	151	31·8 (8·17)	755	32·5 (8·72)
12 mo	226	20·4 (13·19)	211	21·4 (13·64)	151	23·8 (12·63)	550	21·4 (13·29)

In the AUC analysis of PHQ-9 scores over 12 months, the benefit of acupuncture and counselling over usual care alone in terms of score reduction seen at 3 months remained when averaging over the 12-month trial period: acupuncture reduced PHQ-9 scores by −1·55 (95% CI −2·41 to −0·70); counselling by −1·50 (95% CI −2·43 to −0·58) (Table 3).

In secondary analyses, PHQ-9 at 3 months was associated with two factors: expectations of counselling (*p* = 0·064) and expectations about the treatment that patients were allocated to (*p* = 0·015). These factors were included as potential mediators of the effect of trial arm in further analyses. Within the acupuncture and counselling arms, additional significant factors were total session time at 3 months (*p*<0·001) and perceived empathy of practitioners (*p*<0·001), which were added as further covariates.

Linear mixed modelling showed reduced PHQ-9 depression in the acupuncture and counselling groups as compared to usual care at 3 and 6 months (Table 3). The scores in the usual care group continued to reduce over time, such that differences were no longer statistically significant at 9 and 12 months. There was no evidence of significant differences between acupuncture and counselling throughout.

Unadjusted mean scores for the BDI-II at baseline and 12 months are shown in Table 2. Results using ANCOVA show that depression scores on the BDI-II were reduced in the acupuncture arm (−2·88, 95% CI −5·68 to −0·08) and counselling arm (−2·74, 95% CI −5·50 to 0·02) to a greater extent than by usual care alone at 12 months, but with no statistically significant differences between the two interventions (Table 4).

**Table 3:** Effect of trial arm on PHQ-9 depression.

Analysis	Month	*n*	Group 1		Group 2		Group Difference			
					
			Mean	SE	Mean	SE	Mean	SE	95% CI	*p*-Value
			Acupuncture		Usual Care					
ANCOVA^a^	3	452	9·8	0·41	12·3	0·58	−2·46	0·636	−3·72 to −1·21	<0·001
Mixed^b^	3	372	9·8	0·27	12·1	0·38	−2·29	0·475	−3·22 to −1·36	–
	6	350	9·6	0·33	11·5	0·46	−1·90	0·569	−3·02 to −0·79	–
	9	348	10·2	0·39	11·1	0·54	−0·83	0·671	−2·15 to 0·49	–
	12	347	9·7	0·45	10·7	0·64	−0·99	0·785	−2·53 to 0·55	–
AUC^c^	3–12	407	10·9	0·25	12·5	0·35	−1·55	0·435	−2·41 to −0·70	–
			Counselling		Usual Care					
ANCOVA	3	453	11·1	0·40	12·8	0·58	−1·73	0·648	−3·00 to −0·45	0·008
Mixed	3	362	10·9	0·28	12·8	0·38	−1·83	0·477	−2·76 to −0·90	–
	6	345	10·4	0·33	12·2	0·47	−1·78	0·576	−2·91 to −0·65	–
	9	332	10·5	0·40	11·8	0·55	−1·26	0·688	−2·61 to 0·08	–
	12	336	10·4	0·47	11·4	0·65	−1·00	0·805	−2·58 to 0·57	–
AUC	3–12	402	11·6	0·28	13·1	0·38	−1·50	0·470	−2·43	−0·58
			Acupuncture		Counselling					
ANCOVA	3	603	10·0	0·41	10·8	0·40	−0·76	0·514	−1·77 to 0·25	0·140
Mixed	3	402	9·5	0·29	9·4	0·32	0·11	0·439	−0·75 to 0·97	–
	6	371	9·6	0·35	9·1	0·38	0·45	0·527	−0·58 to 1·49	–
	9	360	10·0	0·41	9·0	0·45	0·97	0·621	−0·25 to 2·19	–
	12	361	9·6	0·48	9·0	0·53	0·59	0·721	−0·82 to 2·01	–
AUC	3–12	531	11·1	0·27	11·2	0·27	−0·06	0·378	−0·81 to 0·68	–

a ANCOVA, effect of trial arm on imputed PHQ-9 at 3 months (adjusting for baseline PHQ-9).

b Mixed, effect of trial arm on PHQ-9 over 12 months, (mixed effects model, including time, trial arm x time, baseline PHQ-9, baseline expectation of counselling and baseline expectation of allocated treatment. For the comparison between acupuncture and counselling, treatment time by 3 months and practitioner's empathy were additionally included.)

c AUC, effect of trial arm on average PHQ-9 depression over 12 months (adjusting for Baseline PHQ-9).

SE, standard error.

### Prescribed Medication

The majority (68·7%) of all patients were taking antidepressants at baseline (Table 5). The use of prescribed antidepressants decreased steadily by an average of 12% over the 12-month study period, the rate of which was comparable between trial arms. Around half (47·9%) of patients were taking analgesics at baseline, which decreased on average to 41·0% over 12 months. Patients in the acupuncture arm showed a marked decrease in the use of analgesics in the first 3 months, which was not sustained following the end of the intervention.

**Table 4:** Effect of trial arm on BDI-II depression.

Analysis	Month	*n*	Group 1		Group 2		Group Difference		
			Mean	SE	Mean	SE	Mean	SE	95% CI
			Acupuncture		Usual Care				
ANCOVA^a^	12	445	22·8	1·34	25·7	1·82	−2·88	1·419	−5·68 to −0·08
			Counselling		Usual Care				
ANCOVA	12	449	22·7	1·47	25·4	1·74	−2·74	1·399	−5·50 to 0·02
			Acupuncture		Counselling				
ANCOVA	12	401	22·5	0·92	21·9	1·02	0·59	1·281	−1·93 to 3·11

a ANCOVA, effect of trial arm on imputed BDI-II at 12 months (adjusting for baseline BDI-II, baseline expectation of counselling and baseline expectation of allocated treatment. For the comparison between acupuncture and counselling, treatment time by 3 months and practitioner's empathy were additionally included.)

**Table 5:** Number of patients taking prescribed medication in the preceding 3 months.

Prescribed Medication	Acupuncture + Usual Care			Counselling + Usual Care			Usual Care			Total		
	*n*	Percent^a^	Change from Baseline	Percent	Percent	Change from Baseline	*n*	Percent	Change from Baseline	*n*	Percent	Change from Baseline
**Antidepressants**												
Baseline	189	62·6%	–	220	72·9%	–	110	72·9%	–	519	68·7%	–
3 mo	147	60·7%	−0·9%	155	69·2%	−3·7%	80	66·7%	−6·2%	382	65·2%	−3·5%
6 mo	126	54·6%	−8·0%	145	65·3%	−7·6%	75	63·6%	−9·3%	346	60·6%	−8·1%
9 mo	123	54·0%	−8·6%	124	60·8%	−12·1%	74	66·1%	−6·8%	321	59·0%	−9·7%
12 mo	121	52·4%	−10·2%	124	58·2%	−14·7%	69	61·6%	−11·3%	314	56·5%	−12·2%
**Analgesics**												
Baseline	147	48·8%	–	126	42·3%	–	86	57·3%	–	359	47·9%	–
3 mo	73	30·8%	−18·0%	93	42·5%	+0·2%	57	48·3%	−9·0%	223	38·9%	−9·0%
6 mo	99	43·4%	−5·4%	84	38·5%	−3·8%	52	46·4%	−10·9%	235	42·1%	−5·8%
9 mo	82	36·8%	−12·0%	71	34·8%	−7·5%	55	50·0%	−7·3%	208	38·7%	−9·2%
12 mo	84	36·8%	−12·0%	87	41·0%	−1·3%	54	49·5%	−7·8%	225	41·0%	−6·9%

a All percentages given out of total number of patients with valid responses.

### Adverse Events

The number of patients experiencing a serious adverse event (SAE) over the 12 months, as judged by a clinician (IW), was 16 (5·3%) of 302, 26 (8·6%) of 302, and nine (6·0%) of 151 from the acupuncture, counselling, and usual care groups, respectively, of whom nine had more than one SAE (range 2–4). No SAEs, including three deaths, were known to be related to treatment. The number of patients experiencing a non-serious adverse event (NSAE) was 56 (18·5%), 47 (15·6%), and 40 (26·5%), respectively, of whom 17 had more than one NSAE (range 2–4).

## Discussion

### Principal Findings

In this first randomised trial of acupuncture and counselling for patients continuing to experience depression in primary care, we found statistically significant benefits at 3 months associated with both interventions when provided alongside usual care. This population can be characterised as having experienced recurring bouts of depression (76% having had four or more episodes), with the first episode on average at age 25, some 19 years previously, and 69% were on anti-depressant medication. Moreover significant benefits were observed over the 12-month period as a whole in an AUC analysis. Over time, the usual care group improved such that by 9 and 12 months the benefits of acupuncture and counselling were no longer significantly better than usual care in our primary outcome measure, the PHQ-9. No serious adverse events related to treatment were reported.

### Strengths and Limitations

This study had a clear and practical research question with an appropriate trial design, namely a pragmatic randomised controlled trial, which modelled closely what would happen if patient referrals to acupuncturists and counsellors were routine. Pragmatic trials have an emphasis on external validity with the intention that findings are generalisable to typical patients and settings. We recruited patients in primary care from those who had consulted with depression and continued to be moderately or severely depressed, thereby excluding patients whose symptoms had been alleviated sufficiently by other treatment. Using a different screening measure (BDI-II) from the one we used for measuring outcome (PHQ-9) had the advantage that we avoided the well-known problems of regression towards the mean that occur when we choose people as being above a cut-off. The natural variation in responses to questionnaires produced a few scores below the cut-off for depression on the PHQ9 measurement, 0.8% of participants being below 5, which would have them classified as not depressed, and 11.5% between 5 and 9, which would be mild depression. This is an inevitable consequence of the difficulty of measuring depression. A single PHQ9 below 5 does not mean that someone is not depressed and a single BDI above 20 does not mean that they are depressed, these scores occurring in the same individuals for six participants. Each of these scores is only an uncertain estimate of the person's position on a continuum.

The attrition between invitation to participate and randomisation at baseline was typical of trials that recruit via primary care databases. Multiple imputation was used to compensate to some degree for the limitations related to the loss of follow-up data. The randomisation into groups provided a control for temporal effects and other factors across all patients that might have influenced outcomes beyond the treatment itself. We provided standardised treatment protocols for the acupuncturists and counsellors that were designed to reflect routine practice, allowing individualisation to match patient variability, while ensuring all practitioners met appropriate standards of qualification and experience. While no attempt was made to standardise usual care, it was however provided to patients in all arms of the trial, with a majority of patients continuing with anti-depressants, and differences between groups in usual care at all time points were minimal (see Tables S4–S7). Accordingly, we can assume that the differences between arms can largely be ascribed to the treatments provided by the acupuncturists and the counsellors.

Pragmatic trials, however, tell us less about which aspects of the interventions might be most or least beneficial. Such trials are not designed to separate out relative contributions from treatment components that are specific or non-specific to the intervention. With regard to two treatment components in the acupuncture and counselling groups that are often considered “non-specific,” namely session time and quality of therapist attention, we found that specific treatment effects remained when these were accounted for. Despite the limitation that a pragmatic approach has in ascribing outcomes to different treatment components, exacerbated by some loss to follow-up, this design nevertheless provides a useful estimate of overall effects of complex interventions, an estimate of most interest to patients, practitioners and providers.

### Relationship to the Literature

In a recent Cochrane review of acupuncture for depression, no studies focussed on moderate to severe depression. The only comparator equivalent to the usual care of our study was a wait list comparator, which involved two trials by the same research team [22]–[23]. The Cochrane review's meta-analysis involving 94 patients in these two trials showed a reduction in standardised mean difference (SMD) of −0·73 (95% CI −1·18 to −0·29). This is a larger effect size than we found, however we need to be cautious in the interpretation of the result, as the patients were less depressed and the numbers analysed were small. Regarding counselling, we found no trial based in primary care that evaluated counselling for moderate to severe depression. From a Cochrane review, there is evidence from six trials of counselling for mild to moderate depression with 772 patients that counselling is more effective than usual care in terms of mental health outcomes over 1 to 6 months (SMD −0·28, 95% CI −0·43 to −0·13). These advantages were not shown to endure over the longer term from 7 to 12 months (SMD −0·09, 95% CI −0·27 to 0·10). Their number-needed-to-treat for short-term benefit was 6, somewhat less than the 10 we found for moderate to severely depressed patients. In another review of psychological interventions, four cognitive behaviour therapy studies based in primary care covering 259 patients with depression found a similar short-term effect (SMD −0·33, 95% CI −0·60 to −0·06) [24]. In our rigorously conducted large-scale trial, we present the first evidence on acupuncture and counselling for patients in primary care who are representative of those continuing to experience symptoms of moderate to severe depression. Both interventions are shown to be effective treatments at 3 months when compared to usual care alone, with evidence that there are benefits over the 12-month period in terms of clinical symptoms.

### Implications for Clinical Practice and Future Research

If treatment success is defined as improvement from a depressed PHQ-9 score (≥10) to a non-depressed score (≤9) with an improvement of at least 50% [25], then 33% of acupuncture patients, 29% of counselling patients, and 18% of usual care patients achieved this. These percentages apply to the total number of patients with a baseline PHQ-9 score of 10 or above and for whom data were available at 3 months. The number-needed-to-treat for one additional treatment success, as defined above, was 7 for acupuncture (95% CI 4·3–17·4) and 10 for counselling (95% CI 5·3–47·3). The patient experience can be further illustrated in terms of “depression free days” [26], an approximate summary measure derived from PHQ-9 cut-off scores averaged over the period between measurements. The mean number of depression free days over 3 months was 34 (95% CI 31–38) for the acupuncture group, 27 (95% CI 24–30) for the counselling group, and 23 (95% CI 19–27) for the usual care group.

Further research into the optimal populations and treatment regimens for acupuncture and counselling is merited. The characteristics of patients one recruits to trials of interventions for depression in primary care will depend on the recruitment methods used. Patients recruited at the point of consultation will be different from those recruited via databases, as we did in this trial, and different again from those with depression who are not consulting at all. In the trial we report here, what was important was that patients had consulted in primary care, continued to be depressed, and were seeking other interventions that might help with their depression. A different recruitment method could be used to assess whether the interventions would be more effective for patients referred at the onset of their first episode of depression, rather than after many episodes, as was the case in the present study in which 76% had had four or more previous episodes.

We recruited patients using the Beck Inventory (BDI-II) with scores greater or equal to 20, which is classified by this measure as “moderate to severe.” However other classifications systems, such as DSM, have different categories of severity. What our trial does not tell us is whether acupuncture or counselling performs better or worse for the more mild forms of depression. Nor do we know whether patients not receiving anti-depressant medication (one third of our sample) do better or not, or whether fewer sessions would be sufficient for mild to moderate depression, or more sessions would improve outcomes for those with severe depression (approximately 60% of our sample). For those randomised to acupuncture, it was usually their first experience of this intervention, whereas many randomised to counselling had received this intervention before; however, it is not clear whether this difference impacted on outcomes.

With regard to the acupuncture provision, there is some evidence from the literature that a uniform combination of points can work for depressed patients [27],[28]; however, a head-to-head trial or large individual patient data meta-analysis would be required to determine whether a uniform approach was more beneficial than an individualised approach.

A full economic analysis is needed to combine the costs and effects of each treatment option to determine the cost-effective treatment. Although no other trial data are available on the costs and effects of acupuncture for these primary care patients, other trials exist for counselling in primary care and, to guide policy, these should be synthesized into cost-effectiveness estimates.

## Conclusion

To our knowledge, our study is the first to rigorously evaluate the clinical and economic impact of acupuncture and counselling for patients in primary care who are representative of those who continue to experience depression in primary care. We have provided evidence that acupuncture versus usual care and counselling versus usual care are both associated with a significant reduction in symptoms of depression in the short to medium term, and are not associated with serious adverse events.

## Supporting Information

Protocol S1 **Trial protocol.**
(PDF)

Table S1 **Baseline characteristics for patients with and without missing data at 3 months.**
(DOC)

Table S2 **Details of the acupuncture treatment based on STRICTA reporting guidelines.**
(DOC)

Table S3 **Details of the competences that underpinned the delivery of counselling.**
(DOC)

Table S4 **Usual care provided: number of patients seeing a health professional and average number of visits (total and specifically for depression) for these patients in the preceding 3 months.**
(DOC)

Table S5 **Usual care provided: number of patients attending hospital accident and emergency departments and number of attendances in the preceding 3 months.**
(DOC)

Table S6 **Usual care provided: number of patients admitted to hospital and number of overnight stays in the preceding 3 months.**
(DOC)

Table S7 **Usual care provided: number of patients paying for private health care & money spent in the preceding 3 months.**
(DOC)

Table S8 **Effect of trial arm on PHQ-9 depression at 3 months and BDI-II depression at 12 months (non-imputed data).**
(DOC)

Text S1 **CONSORT statement checklist.**
(DOC)

## Abbreviations

ANCOVA: analysis of covariance
AUC: area under the curve
SD: standard deviation
