# Supplementary material for: Acupuncture and Counselling for Depression in Primary Care: A Randomised Controlled Trial
Source: PLoS Med. 2013 Sep 24;10(9):e1001518. doi: 10.1371/journal.pmed.1001518 (PMC3782410; doi:10.1371/journal.pmed.1001518)
Supplement: Table S1 — Baseline characteristics for patients with and without missing data at 3 months. (DOC) [file pmed.1001518.s002.doc]

**Table S1:** Baseline characteristics for patients with and without missing data at 3 months

| **Characteristic** | **Patients with available data at 3 months**  **N = 616** | | **Patients with missing data at 3 months**  **N = 139** | | **Total**  **N = 755** | |
| --- | --- | --- | --- | --- | --- | --- |
| **Age** |  | |  | |  | |
| Mean (SD) | 44·6 (13·01) | | 38·2 (13·42) | | 43·5 (13·37) | |
| Median (min, max) | 43·5 (18-89) | | 35·0 (18-93) | | 43 (18-93) | |
| Interquartile Range | 35-54 | | 27-46 | | 33-53 | |
| Missing | - | | - | | - | |
| **Sex** |  | |  | |  | |
| Male | 164 (26·6%) | | 37 (26·6%) | | 201 (26·6%) | |
| Female | 452 (73·4%) | | 102 (73·4%) | | 554 (73·4%) | |
| Missing | - | | - | | - | |
| **Depression** |  |  |  |  |  |  |
| In last 2 weeks | 457 | 74·2% | 117 | 84·2% | 574 | 76·0% |
| Missing | 11 | 1·8% | 1 | 0·7% | 12 | 1·6% |
| Not first major episode | 408 | 89·39% | 105 | 89·7% | 513 | 89·4% |
| Missing | 8 | 1·8% | 1 | 0·9% | 9 | 1·6% |
| 4+ previous episodes | 309 | 75·7% | 80 | 76·2% | 389 | 75·8% |
| Missing | 4 | 1·0% | 0 | 0·0% | 4 | 0·8% |
| **Age at first major depressive episode** |  | |  | |  | |
| Mean (SD) | 25·7 (12·5) | | 22·9 (10·8) | | 25·2 (12·28) | |
| Median (min, max) | 22 (0-79) | | 20 (3-78) | | 22 (0-79) | |
| Interquartile Range | 16·5-32 | | 15-30 | | 16-31 | |
| Missing | 12 | 1·9% | 4 | 2·9% | 16 | 2·1% |
| **Medication** |  |  |  |  |  |  |
| Depression medication in last 3 months | 426 | 69·2% | 93 | 66·9% | 519 | 68·7% |
| Missing | 0 | 0·0% | 0 | 0·0% | 0 | 0·0% |
| Analgesic medication in last 3 months | 301 | 48·9% | 58 | 41·7% | 359 | 47·5% |
| Missing | 4 | 0·7% | 2 | 1·4% | 6 | 0·8% |
| **EQ-5D Anxiety/Depression** |  |  |  |  |  |  |
| Not anxious/depressed | 17 | 2·8% | 4 | 2·9% | 21 | 2·8% |
| Moderately anxious/depressed | 465 | 75·5% | 89 | 64·0% | 554 | 73·4% |
| Extremely anxious/depressed | 132 | 21·4% | 46 | 33·1% | 178 | 23·6% |
| Missing | 2 | 0·3% | 0 | 0·0% | 2 | 0·3% |
| **PHQ-9** |  | |  | |  | |
| Mean (SD) | 15·7 (5·32) | | 17·3 (4·94) | | 16·0 (5·29) | |
| Median (min, max) | 16 (3-27) | | 17 (6-27) | | 16 (3-27) | |
| Interquartile Range | 12-20 | | 14-21 | | 12-20 | |
| Missing | 1 | 0·2% | 0 | 0·0% | 1 | 0·1% |
| **PHQ-9 Group** |  |  |  |  |  |  |
| None (0-4) | 6 | 1·0% | 0 | 0·0% | 6 | 0·8% |
| Mild (5-9) | 78 | 12·7% | 9 | 6·5% | 87 | 11·5% |
| Moderate (10-14) | 182 | 29·6% | 35 | 25·2% | 217 | 28·7% |
| Moderately severe (15-19) | 184 | 29·9% | 47 | 33·8% | 231 | 30·6% |
| Severe (20-27) | 165 | 26·8% | 48 | 34·5% | 213 | 28·2% |
| Missing | 1 | 0·2% | 0 | 0·0% | 1 | 0·1% |
| **BDI-II** |  | |  | |  | |
| Mean (SD) | 32·0 (8·62) | | 34·5 (8·91) | | 32·5 (8·72) | |
| Median (min, max) | 31 (20-60) | | 34 (20-53) | | 31 (20-60) | |
| Interquartile Range | 25-37·5 | | 28-41 | | 26-38 | |
| Missing | 0 | 0·0% | 0 | 0·0% | 0 | 0·0% |
| **BDI-II Group** |  |  |  |  |  |  |
| Moderate (20-28) | 248 | 40·3% | 36 | 25·9% | 284 | 37·6% |
| Severe (29-63) | 368 | 59·7% | 103 | 74·1% | 471 | 62·4% |
| Missing | 0 | 0·0% | 0 | 0·0% | 0 | 0·0% |
| **SF-36 Bodily Pain** |  | |  | |  | |
| Mean (SD) | 57·2 (28·54) | | 59·6 (28·01) | | 57·6 (28·44) | |
| Median (min, max) | 56·5 (0-100) | | 52 (0-100) | | 52 (0-100) | |
| Interquartile Range | 31-82 | | 41-84 | | 32-84 | |
| Missing | 4 | 0·6% | 0 | 0·0% | 4 | 0·5% |
| **Treatment Preference** |  |  |  |  |  |  |
| Acupuncture | 343 | 55·7% | 87 | 62·6% | 430 | 57·0% |
| Counselling | 137 | 22·2% | 27 | 19·4% | 164 | 21·7% |
| Usual Care | 10 | 1·6% | 0 | 0·0% | 10 | 1·3% |
| No preference | 122 | 19·8% | 22 | 15·8% | 144 | 19·1% |
| Missing | 4 | 0·7% | 3 | 2·2% | 7 | 0·9% |
