# Supplementary material for: Acupuncture and Counselling for Depression in Primary Care: A Randomised Controlled Trial
Source: PLoS Med. 2013 Sep 24;10(9):e1001518. doi: 10.1371/journal.pmed.1001518 (PMC3782410; doi:10.1371/journal.pmed.1001518)
Supplement: Table S2 — Details of the acupuncture treatment based on STRICTA reporting guidelines. (DOC) [file pmed.1001518.s003.doc]

Table S2: Details of the acupuncture treatment based on the reporting guidelines of STRICTA^i^, an official extension to CONSORT

| **STRICTA item** | **Details of acupuncture treatment within the trial (n=266)** |
| --- | --- |
|  |  |
| 1. a) Style | Traditional Chinese Medicine (TCM). |
| b) Reasoning for treatment provided | Systematic Cochrane review^ii^ and consensus methods^iii^ were used to develop a treatment protocol in a pragmatic trial designed to evaluate acupuncture as provided in routine care. |
| c) Variation | Individualized treatments using common theoretical frameworks of TCM as reported separately. |
| 2. a) Number of needles per treatment | On average 13 needles were inserted per session (range: 3-26). |
| b) Names | 246 different points were used. Common points were SP-6, LIV-3, ST-36, LI-4, which were used within a course of treatment on 91%, 89%, 83% and 74% of patients respectively. |
| c) Depth of insertion | Range: Range of minimum mean depths was 0·49cm, maximum 1·41cm. Total range: 0·05cm to 2·5 cm. |
| d) Response sought | The response sought varied, most commonly *de qi* by 96% of acupuncturists*.* |
| e) Needle stimulation | Manual methods: tonifying (68%), reducing (43%) and even method (55%). |
| f) Retention | Average 22·5 minutes (range: 15 – 45 minutes). |
| g) Needle type | Needle length ranged from 5 to 40mm, and needle diameter ranged from 0·13 to 0·35mm. |
| 3. a) Number of sessions | Patients were offered 12 sessions and completed an average of 10·3 sessions (median 12, range 1 to 12). |
| b) Frequency & duration | Average number of sessions per week, 0·74. Average number of weeks between sessions, 1·36. Average duration of time of session, 53 minutes (range 28 – 95 minutes). |
| 4. a) Other components of treatment | Acupuncturists were allowed to use moxa, electro-acupuncture, ear seeds, cupping, acupressure and heat lamps. Most commonly used were acupressure (used with 13% of patients), moxa (12%) and electro-acupuncture (7%). Acupuncturists were allowed to provide acupuncture theory based lifestyle advice. In total 66% of patients received lifestyle advice, most commonly diet (42%), exercise (30%) and relaxation (25%). Advice unrelated to acupuncture theory as well as herbs and magnets were proscribed. |
| b) Setting and context | Provision or treatments in independent clinics. Acupuncturists encouraged to practice as closely as possible as they normally would, and 96% reported applying acupuncture exactly the same way or similarly as they did routinely. |
| 5. Participating acupuncturists | British Acupuncture Council members, with more than three years post-qualification experience. |
| 6. Control or comparator interventions | Patients continued to receive usual care as an adjunct from their general practitioner, as well as over-the-counter treatments according to need, based on a need to evaluate the impact of acupuncture plus usual care vs. usual care alone. A summary of the usual care received is reported in Table B below. |
|  |  |
| ^i^MacPherson H, Altman DG, et al. Revised STandards for Reporting Interventions in Clinical Trials of Acupuncture (STRICTA): extending the CONSORT statement. PLoS Med 2010 Jun;7(6):e1000261. | |
| ^ii^Smith CA, Hay PP, MacPherson H. Acupuncture for depression. Cochrane Database Syst Rev 2010;(1):CD004046 | |
| ^iii^MacPherson H, Schroer S. Acupuncture as a complex intervention for depression: a consensus method to develop a standardised treatment protocol for a randomised controlled trial. Complement Ther Med 2007 Jun;15(2):92-100. | |
