# Supplementary material for: Acupuncture and Counselling for Depression in Primary Care: A Randomised Controlled Trial
Source: PLoS Med. 2013 Sep 24;10(9):e1001518. doi: 10.1371/journal.pmed.1001518 (PMC3782410; doi:10.1371/journal.pmed.1001518)
Supplement: Table S3 — Details of the competences that underpinned the delivery of counselling. (DOC) [file pmed.1001518.s004.doc]

**Table S3: Details of the competences that underpinned the delivery of counselling**

The counselling was designed to be humanistic in approach and based on relevant competences. These competences were developed independently by Anthony D. Roth, Andrew Hill and Stephen Pilling (2009)^iv^, as commissioned by Skills for Health, supported by an Expert Reference Group and now documented by the Centre for Outcomes Research an Effectiveness at the University College London.^v^. A manualised protocol was developed using an evidence-based methodology. These competences for the delivery of high-quality humanistic psychological therapies covered the following three domains:

1. **Basic competences in humanistic psychological therapies.** Humanistic counselling interventions are underpinned by a range of more specific humanistic techniques. These basic competences set out a range of activities that humanistically-oriented therapists should be able to acknowledge as fundamental to their practice and should be delivered to clients within the trial.
2. **Metacompetences.** These are the competences needed to implement higher-order links between theory and practice and will adapt the implementation of the humanistic approach to the needs of individual clients. These competences are more abstract than those in other domains however they will guide the procedures and practice used by counsellors within the trial.
3. **Specific Humanistic Psychological Therapies competences.** These are technical interventions employed by some (though not all) forms of humanistic psychological therapies. These include for example competences related to approaches to working with emotions and emotional meaning.

^iv^Roth AD, Hill A, Pilling S. The competences required to deliver effective Humanistic Psychological Therapies. University College London, Centre for Outcomes Research and Effectiveness; 2009.

^v^Centre for Outcomes Research & Effectiveness (Core). Humanistic Psychological Therapies Competences Framework [Internet]. UCL Division of Psychology and Language Sciences; 2013 May. Available from: http://www.ucl.ac.uk/clinical-psychology/CORE/humanistic_framework.htm# , 2009) https://tools.skillsforhealth.org.uk/competence_search/?search=humanistic
