# Supplementary material for: Acupuncture and Counselling for Depression in Primary Care: A Randomised Controlled Trial
Source: PLoS Med. 2013 Sep 24;10(9):e1001518. doi: 10.1371/journal.pmed.1001518 (PMC3782410; doi:10.1371/journal.pmed.1001518)
Supplement: Table S4 — Usual care provided: number of patients seeing a health professional and average number of visits (total and specifically for depression) for these patients in the preceding 3 months. (DOC) [file pmed.1001518.s005.doc]

**Table S4: Usual care provided: Number of patients seeing a health professional & average number of visits (total and specifically for depression) for these patients in the preceding three months**

|  |  | **Acupuncture + Usual Care** | | | | **Counselling + Usual Care** | | | | **Usual Care** | | | | **Total** | | | |
| --- | --- | --- | --- | --- | --- | --- | --- | --- | --- | --- | --- | --- | --- | --- | --- | --- | --- |
|  |  | **Patients** | | **Mean number of visits** | | **Patients** | | **Mean number of visits** | | **Patients** | | **Mean number of visits** | | **Patients** | | **Mean number of visits** | |
|  |  | **n** | **%** | **Total** | Depr^1^ | **n** | **%** | **Total** | **Depr** | **n** | **%** | **Total** | **Depr** | **n** | **%** | **Total** | **Depr** |
| GP | 3 months^2^ | 106 | 45·1% | - | 2·2 | 108 | 50·2% | - | 2·3 | 70 | 58·8% | - | 2·2 | 284 | 49·9% | - | 2·2 |
|  | 6 months | 153 | 73·6% | 2·8 | 0·8 | 134 | 68·0% | 2·5 | 0·8 | 73 | 74·5% | 2·6 | 1·1 | 360 | 71·6% | 2·7 | 0·9 |
|  | 9 months | 135 | 67·2% | 2·2 | 0·7 | 121 | 66·5% | 2·2 | 0·8 | 81 | 75·7% | 2·3 | 0·9 | 337 | 68·8% | 2·3 | 0·8 |
|  | 12 months | 127 | 63·2% | 2·4 | 1·0 | 131 | 68·2% | 2·3 | 0·7 | 73 | 69·5% | 2·4 | 0·7 | 331 | 66·5% | 2·4 | 0·8 |
| Practice Nurse | 3 months | 7 | 3·7% | - | 1·4 | 12 | 6·9% | - | 2·0 | 8 | 9·1% | - | 1·8 | 27 | 6·3% | - | 1·8 |
|  | 6 months | 58 | 26·1% | 1·7 | 0·0 | 63 | 29·9% | 1·5 | 0·1 | 36 | 33·0% | 1·4 | 0·0 | 157 | 29·0% | 1·6 | 0·0 |
|  | 9 months | 55 | 25·0% | 1·7 | 0·1 | 50 | 25·1% | 1·9 | 0·0 | 28 | 25·5% | 1·8 | 0·3 | 133 | 25·1% | 1·8 | 0·1 |
|  | 12 months | 54 | 25·0% | 1·6 | 0·0 | 63 | 30·7% | 1·4 | 0·0 | 39 | 35·5% | 1·8 | 0·0 | 156 | 29·4% | 1·6 | 0·0 |
| Outpatient Clinic | 3 months | 11 | 4·6% | - | 2·0 | 13 | 5·9% | - | 2·1 | 9 | 7·4% | - | 3·3 | 33 | 5·7% | - | 2·6 |
|  | 6 months | 46 | 20·6% | 2·3 | 0·1 | 60 | 28·4% | 2·2 | 0·1 | 25 | 23·1% | 1·3 | 0·0 | 131 | 24·2% | 2·1 | 0·1 |
|  | 9 months | 50 | 22·4% | 1·8 | 0·2 | 44 | 22·3% | 2·0 | 0·2 | 27 | 25·2% | 1·5 | 0·1 | 121 | 23·0% | 1·8 | 0·2 |
|  | 12 months | 61 | 28·1% | 1·7 | 0·3 | 57 | 28·4% | 1·8 | 0·2 | 35 | 32·4% | 1·7 | 0·1 | 153 | 29·1% | 1·8 | 0·2 |
| Mental Health Nurse | 3 months | 5 | 2·2% | - | 1·6 | 1 | 0·5% | - | 5·0 | 4 | 3·5% | - | 4·5 | 10 | 1·8% | - | 3·1 |
|  | 6 months | 4 | 1·8% | 4·0 | 2·3 | 1 | 0·5% | 8·0 | - | 2 | 1·8% | 3·5 | 3·5 | 7 | 1·3% | 4·4 | 2·8 |
|  | 9 months | 7 | 3·2% | 4·1 | 2·9 | 3 | 1·5% | 5·3 | 6·5 | 4 | 3·8% | 3·0 | 1·8 | 14 | 2·7% | 4·1 | 3·1 |
|  | 12 months | 11 | 5·1% | 3·5 | 2·3 | 4 | 2·1% | 2·3 | 1·8 | 3 | 2·9% | 4·0 | 2·3 | 18 | 3·5% | 3·3 | 2·2 |
| Psychologist or Psychiatrist | 3 months | 4 | 1·8% | - | 1·8 | 5 | 2·4% | - | 6·2 | 6 | 5·4% | - | 2·7 | 15 | 2·7% | - | 3·6 |
|  | 6 months | 10 | 4·5% | 1·7 | 1·2 | 9 | 4·5% | 4·7 | 1·7 | 7 | 6·5% | 2·9 | 1·6 | 26 | 4·9% | 3·0 | 1·5 |
|  | 9 months | 7 | 3·3% | 2·3 | 2·1 | 7 | 3·6% | 1·9 | 1·3 | 4 | 3·8% | 5·5 | 5·3 | 18 | 3·5% | 2·8 | 2·5 |
|  | 12 months | 11 | 5·1% | 3·5 | 3·5 | 7 | 3·7% | 2·9 | 2·0 | 5 | 4·8% | 2·2 | 1·4 | 23 | 4·5% | 3·0 | 2·6 |
| NHS Counsellor | 3 months | 5 | 2·2% | - | 3·0 | 4 | 1·9% | - | 3·0 | 5 | 4·4% | - | 2·4 | 14 | 2·5% | - | 2·8 |
|  | 6 months | 9 | 4·2% | 2·7 | 1·0 | 5 | 2·5% | 3·2 | 3·8 | 5 | 4·8% | 1·4 | 0·5 | 19 | 3·6% | 2·5 | 1·6 |
|  | 9 months | 5 | 2·3% | 3·0 | 1·8 | 2 | 1·0% | 3·5 | 3·5 | 4 | 3·9% | 3·8 | 3·0 | 11 | 2·1% | 3·4 | 2·6 |
|  | 12 months | 10 | 4·7% | 3·8 | 3·1 | 3 | 1·5% | 3·3 | 4·0 | 5 | 5·0% | 2·2 | 2·2 | 18 | 3·5% | 3·3 | 2·9 |
| Other NHS health professional | 3 months | 16 | 8·9% | - | 3·9 | 10 | 6·1% | - | 2·5 | 15 | 19·0% | - | 3·1 | 41 | 9·7% | - | 3·2 |
|  | 6 months | 20 | 9·1% | 2·3 | 0·3 | 26 | 12·6% | 2·7 | 0·0 | 14 | 13·3% | 2·1 | 0·0 | 60 | 11·3% | 2·4 | 0·1 |
|  | 9 months | 24 | 11·2% | 2·8 | 0·6 | 26 | 13·3% | 2·5 | 0·3 | 16 | 14·5% | 2·2 | 0·5 | 66 | 12·7% | 2·5 | 0·4 |
|  | 12 months | 25 | 11·5% | 2·3 | 0·5 | 32 | 16·2% | 2·7 | 0·0 | 20 | 18·7% | 3·1 | 0·2 | 77 | 14·8% | 2·7 | 0·2 |
| ^1^Depr = Specifically for depression (out of total) | | | | | | | | | | | | | | | | | |
| ^2^Questions at 3 months were phrased differently from following time points, asking for responses in relation to depression only | | | | | | | | | | | | | | | | | |
