# Supplementary material for: Acupuncture and Counselling for Depression in Primary Care: A Randomised Controlled Trial
Source: PLoS Med. 2013 Sep 24;10(9):e1001518. doi: 10.1371/journal.pmed.1001518 (PMC3782410; doi:10.1371/journal.pmed.1001518)
Supplement: Table S5 — Usual care provided: number of patients attending hospital accident and emergency departments and number of attendances in the preceding 3 months. (DOC) [file pmed.1001518.s006.doc]

## Table S5: Usual care provided: Number of patients attending A&E and number of attendances in the preceding three months

|  | **Acupuncture + Usual Care** | | | | **Counselling + Usual Care** | | | | **Usual Care** | | | | **Total** | | | |
| --- | --- | --- | --- | --- | --- | --- | --- | --- | --- | --- | --- | --- | --- | --- | --- | --- |
|  | **Patients** | | **Number of attendances** | | **Patients** | | **Number of attendances** | | **Patients** | | **Number of attendances** | | **Patients** | | **Number of attendances** | |
|  | **n** | **%** | **Mean** | **SD** | **n** | **%** | **Mean** | **SD** | **n** | **%** | **Mean** | **SD** | **n** | **%** | **Mean** | **SD** |
| 3 months | 1 | 0·4% | 3·0 | - | 3 | 1·3% | 1·7 | 1·15 | 3 | 2·4% | 1·3 | 0·58 | 7 | 1·2% | 1·7 | 0·95 |
| 6 months | 17 | 7·5% | 1·1 | 0·33 | 20 | 8·8% | 1·8 | 2·51 | 11 | 9·7% | 1·1 | 0·30 | 48 | 8·5% | 1·4 | 1·65 |
| 9 months | 11 | 4·8% | 1·3 | 0·47 | 13 | 6·3% | 1·4 | 0·96 | 12 | 10·7% | 1·5 | 1·00 | 36 | 6·6% | 1·4 | 0·84 |
| 12 months | 21 | 9·2% | 1·2 | 0·40 | 11 | 5·3% | 1·3 | 0·47 | 12 | 10·5% | 1·1 | 0·29 | 44 | 8·0% | 1·2 | 0·39 |
