# Supplementary material for: Acupuncture and Counselling for Depression in Primary Care: A Randomised Controlled Trial
Source: PLoS Med. 2013 Sep 24;10(9):e1001518. doi: 10.1371/journal.pmed.1001518 (PMC3782410; doi:10.1371/journal.pmed.1001518)
Supplement: Table S6 — Usual care provided: number of patients admitted to hospital and number of overnight stays in the preceding 3 months. (DOC) [file pmed.1001518.s007.doc]

**Table S6: Usual care provided: Number of patients admitted to hospital & number of overnight stays in the preceding 3 months**

|  | **Acupuncture + Usual Care** | | | | **Counselling + Usual Care** | | | | **Usual Care** | | | | **Total** | | | |
| --- | --- | --- | --- | --- | --- | --- | --- | --- | --- | --- | --- | --- | --- | --- | --- | --- |
|  | **Patients** | | **Number of overnight stays** | | **Patients** | | **Number of overnight stays** | | **Patients** | | **Number of overnight stays** | | **Patients** | | **Number of overnight stays** | |
|  | **n** | **%** | **Mean** | **SD** | **n** | **%** | **Mean** | **SD** | **n** | **%** | **Mean** | **SD** | **n** | **%** | **Mean** | **SD** |
| **Hospital Ward** |  |  |  |  |  |  |  |  |  |  |  |  |  |  |  |  |
| 3 months | 2 | 0·8% | 3·5 | 0·71 | 0 | 0·0% | - | - | 0 | 0·0% | - | - | 2 | 0·3% | 3·5 | 0·71 |
| 6 months | 6 | 2·8% | 2·0 | 0·89 | 10 | 4·8% | 5·6 | 6·59 | 2 | 1·8% | 6·0 | 1·41 | 18 | 3·4% | 4·4 | 5·15 |
| 9 months | 4 | 1·9% | 3·8 | 3·20 | 4 | 2·0% | 6·8 | 4·79 | 7 | 6·1% | 3·1 | 3·76 | 15 | 2·9% | 4·3 | 3·95 |
| 12 months | 6 | 2·7% | 2·0 | 0·89 | 7 | 3·4% | 1·9 | 1·46 | 8 | 7·1% | 2·5 | 2·00 | 21 | 3·9% | 2·1 | 1·53 |
| **Intensive Care** |  |  |  |  |  |  |  |  |  |  |  |  |  |  |  |  |
| 3 months | 0 | 0·0% | - | - | 0 | 0·0% | - | - | 0 | 0·0% | - | - | 0 | 0·0% | - | - |
| 6 months | 0 | 0·0% | - | - | 1 | 0·5% | 2·0 | - | 0 | 0·0% | - | - | 1 | 0·2% | 2·0 | - |
| 9 months | 0 | 0·0% | - | - | 1 | 0·5% | 4·0 | - | 0 | 0·0% | - | - | 1 | 0·2% | 4·0 | - |
| 12 months | 1 | 0·5% | 6·0 | - | 0 | 0·0% | - | - | 0 | 0·0% | - | - | 1 | 0·2% | 6·0 | - |
| **Mental Health Unit** |  |  |  |  |  |  |  |  |  |  |  |  |  |  |  |  |
| 3 months | 0 | 0·0% | - | - | 0 | 0·0% | - | - | 1 | 0·8% | 1·0 | - | 1 | 0·2% | 1·0 | - |
| 6 months | 1 | 0·5% | 7·0 | - | 1 | 0·5% | 3·0 | - | 0 | 0·0% | - | - | 2 | 0·4% | 5·0 | 2·82 |
| 9 months | 0 | 0·0% | - | - | 1 | 0·5% | 3·0 | - | 1 | 0·9% | 18·0 | - | 2 | 0·4% | 10·5 | 10·61 |
| 12 months | 0 | 0·0% | - | - | 1 | 0·5% | 2·0 | - | 1 | 0·9% | 21·0 | - | 2 | 0·4% | 11·5 | 13·44 |
| **Other** |  |  |  |  |  |  |  |  |  |  |  |  |  |  |  |  |
| 3 months | 0 | 0·0% | - | - | 1 | 0·5% | 3·0 | - | 1 | 0·8% | 1·0 | - | 2 | 0·3% | 2·0 | 1·41 |
| 6 months | 0 | 0·0% | - | - | 1 | 0·5% | 1·0 | - | 0 | 0·0% | - | - | 1 | 0·2% | 1·0 | - |
| 9 months | 2 | 0·9% | 3·5 | 3·54 | 3 | 1·5% | 1·0 | 0·00 | 0 | 0·0% | - | - | 5 | 1·0% | 2·0 | 2·24 |
| 12 months | 1 | 0·5% | 7·0 | - | 1 | 0·5% | 1·0 | - | 0 | 0·0% | - | - | 2 | 0·4% | 4·0 | 4·24 |
