# Supplementary material for: Acupuncture and Counselling for Depression in Primary Care: A Randomised Controlled Trial
Source: PLoS Med. 2013 Sep 24;10(9):e1001518. doi: 10.1371/journal.pmed.1001518 (PMC3782410; doi:10.1371/journal.pmed.1001518)
Supplement: Table S7 — Usual care provided: number of patients paying for private health care & money spent in the preceding 3 months. (DOC) [file pmed.1001518.s008.doc]

**Table S7: Usual care provided: Number of patients paying for private health care & money spent in the preceding 3 months**

|  | **Acupuncture + Usual Care** | | | | **Counselling + Usual Care** | | | | **Usual Care** | | | | **Total** | | | |
| --- | --- | --- | --- | --- | --- | --- | --- | --- | --- | --- | --- | --- | --- | --- | --- | --- |
|  | **Patients** | | **£ spent** | | **Patients** | | **£ spent** | | **Patients** | | **£ spent** | | **Patients** | | **£ spent** | |
|  | **n** | **%** | **Mean** | **SD** | **n** | **%** | **Mean** | **SD** | **n** | **%** | **Mean** | **SD** | **n** | **%** | **Mean** | **SD** |
| **Private Acupuncture** |  |  |  |  |  |  |  |  |  |  |  |  |  |  |  |  |
| 3 months | 3 | 1·2% | 60·0 | 21·79 | 1 | 0·4% | 300·0 | - | 2 | 1·6% | 117·5 | 116·67 | 6 | 1·0% | 119·2 | 107·49 |
| 6 months | 27 | 11·5% | 96·8 | 73·29 | 3 | 1·3% | 146·7 | 80·83 | 3 | 2·5% | 63·3 | 49·33 | 33 | 5·7% | 98·4 | 72·48 |
| 9 months | 27 | 11·6% | 91·5 | 68·32 | 2 | 0·9% | 130·0 | 70·71 | 2 | 1·7% | 75·0 | 63·64 | 31 | 5·5% | 93·0 | 66·68 |
| 12 months | 22 | 9·6% | 108·1 | 68·75 | 2 | 0·9% | 129·5 | 43·13 | 2 | 1·7% | 75·0 | 63·64 | 26 | 4·6% | 107·2 | 65·80 |
| **Private Counselling** |  |  |  |  |  |  |  |  |  |  |  |  |  |  |  |  |
| 3 months | 2 | 0·8% | 162·5 | 53·03 | 3 | 1·3% | 453·3 | 646·71 | 2 | 1·6% | 152·5 | 137·89 | 7 | 1·2% | 284·3 | 409·96 |
| 6 months | 4 | 1·7% | 116·7 | 57·74 | 13 | 5·8% | 198·5 | 103·35 | 1 | 0·8% | 130·0 | - | 18 | 3·1% | 180·0 | 98·04 |
| 9 months | 0 | 0·0% | - | - | 10 | 4·7% | 162·5 | 112·18 | 0 | 0·0% | - | - | 10 | 1·8% | 162·5 | 112·18 |
| 12 months | 3 | 1·3% | 175·0 | 238·48 | 10 | 4·6% | 170·8 | 107·65 | 1 | 0·9% | 65·0 | - | 14 | 2·5% | 164·1 | 132·63 |
| **Psychotherapy** |  |  |  |  |  |  |  |  |  |  |  |  |  |  |  |  |
| 3 months | 0 | 0·0% | - | - | 7 | 3·2% | 175·7 | 218·26 | 2 | 1·9% | 40·0 | - | 9 | 1·6% | 158·8 | 207·69 |
| 6 months | 0 | 0·0% | - | - | 2 | 0·9% | 152·5 | 67·18 | 1 | 0·8% | 160·0 | - | 3 | 0·5% | 155·0 | 47·70 |
| 9 months | 0 | 0·0% | - | - | 2 | 1·0% | 291·5 | 362·75 | 0 | 0·0% | - | - | 2 | 0·4% | 291·5 | 362·75 |
| 12 months | 1 | 0·4% | 450·0 | - | 2 | 0·9% | 182·5 | 166·17 | 1 | 0·9% | 65·0 | - | 4 | 0·7% | 220·0 | 189·16 |
