# Supplementary material for: Acupuncture and Counselling for Depression in Primary Care: A Randomised Controlled Trial
Source: PLoS Med. 2013 Sep 24;10(9):e1001518. doi: 10.1371/journal.pmed.1001518 (PMC3782410; doi:10.1371/journal.pmed.1001518)
Supplement: Table S8 — Effect of trial arm on PHQ-9 depression at 3 months and BDI-II depression at 12 months (non-imputed data). (DOC) [file pmed.1001518.s009.doc]

**Table S8**: Effect of trial arm on PHQ-9 depression at 3 months and BDI-II depression at 12 months (non-imputed data)

| **Analysis** | **N** | **Group 1** | | **Group 2** | | **Group difference** | | | | | |
| --- | --- | --- | --- | --- | --- | --- | --- | --- | --- | --- | --- |
|  |  | **Mean** | **SE** | **Mean** | **SE** | **Mean** | **SE** | **95% CI** | | | **p** |
|  |  | Acupuncture | | Usual Care | |  |  |  |  |  |  |
| PHQ-9 ^1^ | 376 | 9·8 | 0·35 | 12·4 | 0·48 | −2·58 | 0·598 | −3·76 | , | −1·40 | <0·001 |
| BDI-II ^2^ | 335 | 21·3 | 1.25 | 24.6 | 1.70 | −3.27 | 1.462 | −6.14 | , | −0.39 | 0.026 |
|  |  | Counselling | | Usual Care | |  |  |  |  |  |  |
| PHQ-9 ^1^ | 365 | 11·0 | 0·38 | 12·9 | 0·52 | −1·97 | 0·639 | −3·22 | , | −0·71 | 0·002 |
| BDI-II ^2^ | 321 | 23.9 | 1.37 | 27.2 | 1.62 | −3.37 | 1.445 | −6.21 | , | −0.52 | 0.021 |
|  |  | Acupuncture | | Counselling | |  |  |  |  |  |  |
| PHQ-9 ^1^ | 485 | 10·0 | 0·36 | 10·7 | 0·37 | −0·65 | 0·516 | −1·67 | , | 0·36 | 0·207 |
| BDI-II ^2^ | 354 | 21.7 | 1.21 | 21.1 | 1.37 | 0.62 | 1.300 | −1.95 | , | 3.18 | 0.636 |

^1^ PHQ-9: Effect of trial arm on non-imputed PHQ-9 at 3 months (Analysis of covariance, adjusting for baseline PHQ-9)

^2^ BDI-II: Effect of trial arm on non-imputed BDI-II at 12 months (Analysis of Covariance, adjusting for baseline BDI-II, baseline expectation of counselling and baseline expectation of allocated treatment. For the comparison between acupuncture and counselling, treatment time by 3 months and practitioner’s empathy were additionally included.)
